# Kinematic Modeling at the Ant Scale: Propagation of Model Parameter Uncertainties

**DOI:** 10.3389/fbioe.2022.767914

**Published:** 2022-03-01

**Authors:** Santiago Arroyave-Tobon, Jordan Drapin, Anton Kaniewski, Jean-Marc Linares, Pierre Moretto

**Affiliations:** ^1^ Institut Des Sciences Du Mouvement, Faculté Des Sciences Du Sport, Aix-Marseille Université, CNRS, Marseille, France; ^2^ Centre de Recherches sur la Cognition Animale (CRCA), Centre de Biologie Intégrative (CBI), Université de Toulouse, CNRS, UPS, Toulouse, France

**Keywords:** multibody, inverse kinematics, ant, motion capture, uncertainty

## Abstract

Quadrupeds and hexapods are known by their ability to adapt their locomotive patterns to their functions in the environment. Computational modeling of animal movement can help to better understand the emergence of locomotive patterns and their body dynamics. Although considerable progress has been made in this subject in recent years, the strengths and limitations of kinematic simulations at the scale of small moving animals are not well understood. In response to this, this work evaluated the effects of modeling uncertainties on kinematic simulations at small scale. In order to do so, a multibody model of a *Messor barbarus* ant was developed. The model was built from 3D scans coming from X-ray micro-computed tomography. Joint geometrical parameters were estimated from the articular surfaces of the exoskeleton. Kinematic data of a free walking ant was acquired using high-speed synchronized video cameras. Spatial coordinates of 49 virtual markers were used to run inverse kinematics simulations using the OpenSim software. The sensitivity of the model’s predictions to joint geometrical parameters and marker position uncertainties was evaluated by means of two Monte Carlo simulations. The developed model was four times more sensitive to perturbations on marker position than those of the joint geometrical parameters. These results are of interest for locomotion studies of small quadrupeds, octopods, and other multi-legged animals.

## 1 Introduction

Legged locomotion is the most common form of terrestrial animal movement (Christensen et al., 2021). Even if quadrupedal and hexapodal forms of locomotion have evolved independently (Blickhan and Full, 1987), they present similarities. Both quadrupeds and hexapods can adapt their locomotive patterns according to their objective (Hoyt and Taylor, 1981; Nirody, 2021). Like quadrupeds, hexapods exhibit a wide variety of locomotor strategies (Nirody, 2021), e.g., walking, running, and jumping (Musthak Ali et al., 1992) or even swimming (Schultheiss and Guénard, 2021) and gliding hovering (Yanoviak et al., 2005). As some quadrupeds do, insects change smoothly the inter-leg coordination patterns based on their locomotion speed (Ambe et al., 2018). In the metachronous gait (or direct wave gait), hexapods propagate swinging movements from the hind legs to the forelegs, similarly as quadrupeds do in the walking gait (Ambe et al., 2018). In tripod gait, hexapods move their diagonal legs in phases, as quadrupeds do in the trotting gait (Ambe et al., 2018). These equivalences in the locomotion mechanics generate similar ground reaction force patterns in quadrupeds and hexapods, as demonstrated experimentally by Full et al. (1991). In that study, the authors demonstrated that at constant average speed, cockroaches function as a spring–mass system in which three legs add up to function as one leg of a biped or two legs of a quadruped.

As opposed to bipedal and quadrupedal locomotion, hexapodal locomotion is characterized by its plasticity. For instance, hexapods can adopt quadrupedal or bipedal gaits to increase speed, as has been shown in cockroaches (Full et al., 1991). The bipedal posture adopted when the insect stands up allows for a longer stride length while maintaining the same stride frequency, thus raising the speed. In stick insects, the coordination of the middle legs and hind legs is similar to the typical regular gait of quadrupeds (Grabowska et al., 2012). The emergence of quadrupedal gaits on hexapod robots has also been demonstrated when a sudden fault event occurs to one leg (Yang and Kim, 1998). However, these adaptations deserve further analysis to better understand the plasticity and dynamics of multi-legged gait.

The hexapodal gait has been first described as an alternative tripod gait that ensures high static stability (Hughes, 1952) regardless of the support. Yet studies estimating ground reaction forces demonstrate different functions of the rear, median, and front legs (sustain, propel, push, or drag) (Cruse, 1976; Full et al., 1991; Grabowska et al., 2012; Reinhardt and Blickhan, 2014; Wöhrl et al., 2017). Other studies, dedicated to the effects of the ground substrates or load carried, demonstrated the plasticity of the tripod gait in response to mechanical constraints (Bernadou et al., 2011; Pfeffer et al., 2019; Merienne et al., 2020). These studies suggest that hexapodal gait is more complex than a mere alternating tripod one. Furthermore, the small scale and lack of a precise description of the architecture of the musculoskeletal system could explain why the hexapodal gait is less documented than the quadrupedal or bipedal gaits.

Learning how insects adapt their locomotion strategies to their environment (motor and neural control), how each body segment moves for a given locomotion strategy (kinematics), and how forces are generated (muscle actuation) and transmitted (joint dynamics) could help answer biological questions and develop engineering applications. For instance, kinematic, dynamic, and motor control data regarding animal locomotion proved indispensable for bio-inspired robotics development. Particularly, some examples of applications include bio-inspired robot architecture (Lu et al., 2018), bio-inspired control strategies for legged robots (Dupeyroux et al., 2019; Ouyang et al., 2021), and bio-inspired actuation systems (Ahn et al., 2019), among others.

Computational modeling of animal movement can help us better understand the emergence of locomotive patterns and their mechanics by means of musculoskeletal models. A musculoskeletal model is composed of a kinematic model coupled to a dynamic model. The kinematic model, which represents the skeletal system, is a set of body segments connected by joints (i.e., a multibody system). A dynamic model, which represents the muscular system, is a set of actuators attached to the skeletal system.

The proper development of the kinematic model is essential for predicting later muscle and joint forces (Dunne et al., 2021). In kinematic modeling, constrained inverse kinematics, as opposed to with unconstrained inverse kinematics, leads to a more realistic prediction of joint kinematics. Conversely, unconstrained inverse kinematics, which permits a fast exploitation of experimental data using stick models, can generate unrealistic behaviors, such as a model’s body segment changing length (Dunne et al., 2021). This kind of behavior is unsuitable for musculoskeletal simulations. In constrained kinematic modeling, which is conducted using multibody models, the position and orientation of each segment of the kinematic chain are derived from the trajectories of experimental markers. This is done by optimizing procedures that minimize the weighted least-squares distance between experimental markers and the corresponding markers placed on the kinematic model (Lu and O’Connor, 1999). The position and orientation of each segment of the kinematic chain, together with their first-order derivatives, can be used for further muscle and joint force estimation.

In the case of vertebrates, the development and use of musculoskeletal models are mainly motivated by medical applications (REFS). In the case of insects, motivations are mostly related to biology, ecology, and evolution. Ramdya et al. (2017) developed a multibody model of *Drosophila* to study fast locomotor gaits. Guo et al. (2018) proposed a neuromusculoskeletal model for insects to study control strategies in gait patterns. David et al. (2016) and Blanke et al. (2017) developed musculoskeletal models of the dragonfly’s mandible to study bite forces. A kinematic model of stick insects was developed by Theunissen and Dürr (2013). In the case of ants, locomotion studies mostly focus on experimental procedures. Examples are video-based kinematic analysis (Weihmann and Blickhan, 2009; Moll et al., 2010; Pfeffer et al., 2019), stepping pattern analysis (Zollikofer, 1994), center of mass tracking (Reinhardt and Blickhan, 2014; Merienne et al., 2020; Merienne et al., 2021), quantification of ground reaction forces (Reinhardt et al., 2009; Wöhrl et al., 2017), and mandible forces (Zhang et al., 2020), among others.

Despite the aforementioned examples, the use of musculoskeletal models at the insect scale is not yet widespread, probably due to the technological barriers to acquire experimental data (kinematic, dynamic, and morphometric data). When we compare the relative resolution of motion capture systems *vs*. the subject size, it can be argued that motion capture at the human scale is far more accurate than at the insect scale. In human motion analysis using reflective markers, the measuring uncertainty can reach 0.33 mm in a volume of 5.5 × 1.2 × 2.0 m^3^ (Eichelberger et al., 2016) (0.0275% in the smallest dimension). Motion analysis by means of physical markers is not easy in small insects. A pattern-matching procedure based on video films is a feasible solution for the moment. With the use of this technique at the small scale, our setup reached, on average, 3% resolution in each dimension of the calibrated volume (including tracking errors and pattern recognition errors). The difficulty with small scales lies in keeping the depth of field of the camera at a reasonable size when zooming in to get a clear whole-body image. This problem is not encountered in larger subjects because the lenses are far from the objective. Similar difficulties are faced in morphometric data acquisition in small insects, which is required for the definition of joint locations in musculoskeletal modeling. This implies that the effect of uncertainties in musculoskeletal modeling at the insect scale must be considered and evaluated to understand the limits of this tool in locomotion analysis. Estimation of uncertainties in kinematic modeling has been widely addressed at the human scale (see, for example, Groen et al., 2012; El Habachi et al., 2015; Martelli et al., 2015). At the insect scale, however, it is unclear how modeling assumptions affect predicted results in kinematic modeling.

The present work therefore evaluated the effects of modeling assumptions in kinematic analysis at the small insect scale, particularly on a *Messor barbarus* ant. To achieve this objective, (1) a whole-body kinematic model of the *Messor barbarus* ant was developed (Section 2.1), (2) an inverse kinematics simulation of the ant gait was reproduced using the developed model and experimental kinematic data (Section 2.6), and (3) the sensitivity of the predicted results regarding model parameter uncertainties was evaluated (Section 2.7).

## 2 Methods

**FIGURE 1:**
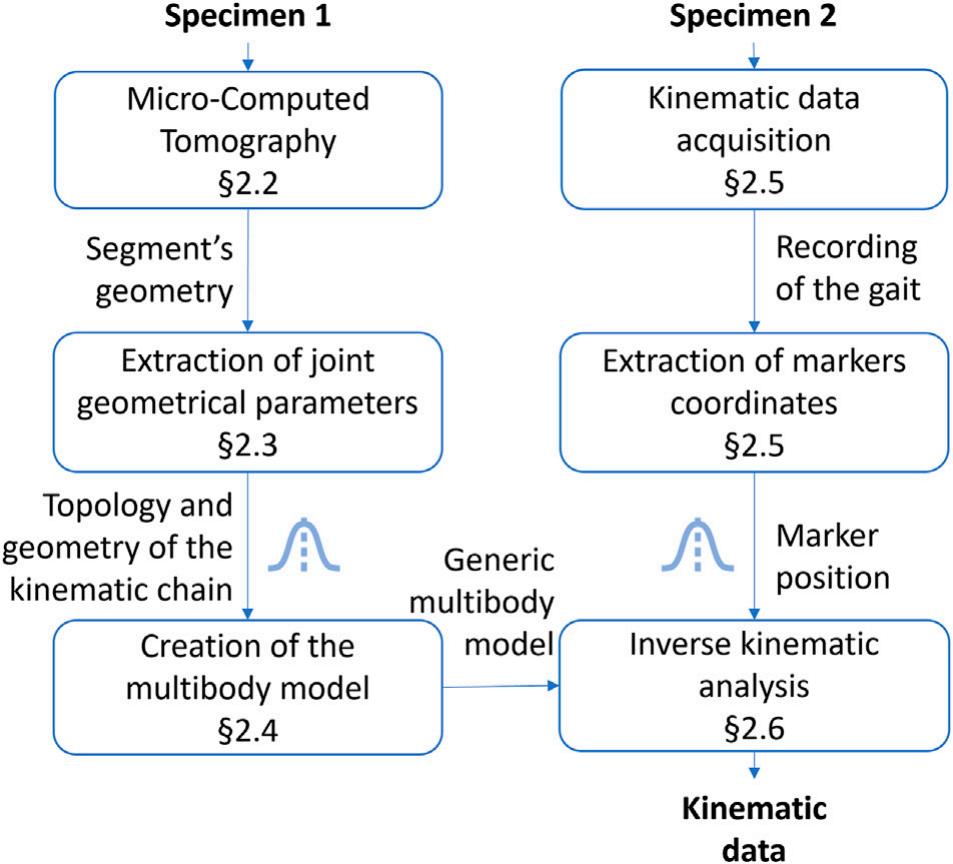
Followed research methodology. The Gaussian distribution icon indicates model parameters subjected to uncertainty.

The global research methodology followed in this work is illustrated in Figure 1. Specimens 1 and 2 belong to the medium-sized caste of the *Messor barbarus* species (more details in Section 2.1). Specimen 1 was used to build a 3D model from micro-computed tomography (Section 2.2). 3D models of body segments were used to extract joint geometrical parameters and to create a multibody model (Section 2.3 and Section 2.4). Specimen 2 was used to acquire experimental kinematic data and to extract marker trajectories (Section 2.5). Experimental kinematic data were used to scale the multibody model and to run an inverse kinematics simulation (Section 2.6). To evaluate the impact of the propagation of model parameter uncertainties on joint angles, two Monte Carlo (MC) simulations were conducted (Section 2.7). Model parameters subjected to uncertainty are represented by a Gaussian distribution icon in Figure 1.

### 2.1 Experimental Model

We used workers from a colony of *Messor barbarus* collected in April 2018 in Saint-Hippolyte (42°78 north; 2°97 east, Pyrénées-Orientales, France). *Messor barbarus* is a seed-collecting ant whose mature colonies can harbor tens of thousands of individuals (Hölldobler and Wilson, 1990). The body mass of the scanned subject was 8.92 mg.

The main colony was kept in a box (L: 50 cm × W: 30 cm × H: 15 cm) with walls coated with Fluon^®^ to prevent ants from escaping. The ants could shelter inside nests formed with test tubes (length: 20 cm; diameter: 2.5 cm) covered with opaque paper. They had access to water and a mixture of bird seeds. The experimental room was maintained at a constant temperature of 26°C (thermometer: TFA Dostmann/Wertheim) and under an artificial photoperiod regime 12 h:12 h (light:dark).

### 2.2 Micro-Computed Tomography

Following the procedure used by Peeters et al. (2020), specimen 1 was stored in 90% ethanol, then stained in a 2 M iodine solution for a minimum of 24 h, and transferred into micro-tubes filled with 99% ethanol. It was then transferred to the Okinawa Institute of Science and Technology Graduate University (OIST, Japan) to be scanned using micro-computed tomography (µ-CT). This was performed using a Zeiss Xradia 510 Versa 3D X-ray microscope operated by the Zeiss Scout-and-Scan Control System software (version 11.1). A vertical stitching enabled a three-times scanning along a head–trunk–gaster axis, each with a resolution of 933 × 1,013 × 988 pixels (providing a voxel of 5.7 µm). These scans were compiled to increase the resolution of the whole ant body to 3,159 × 1,013 × 988 pixels. The DICOM images of the µ-CT scan were used to build the 3D models of the body segments. A segmentation was done using ITK-SNAP (version 3.6.0) (Yushkevich et al., 2006) to differentiate the body segments as follows: head, thorax, abdomen, coxa, trochanter, femur, tibia, metatarsus, and tarsus. The four tarsal segments were lumped all into a unique rigid segment called tarsus in this work.

### 2.3 Extraction of Joint Geometrical Parameters

**FIGURE 2:**
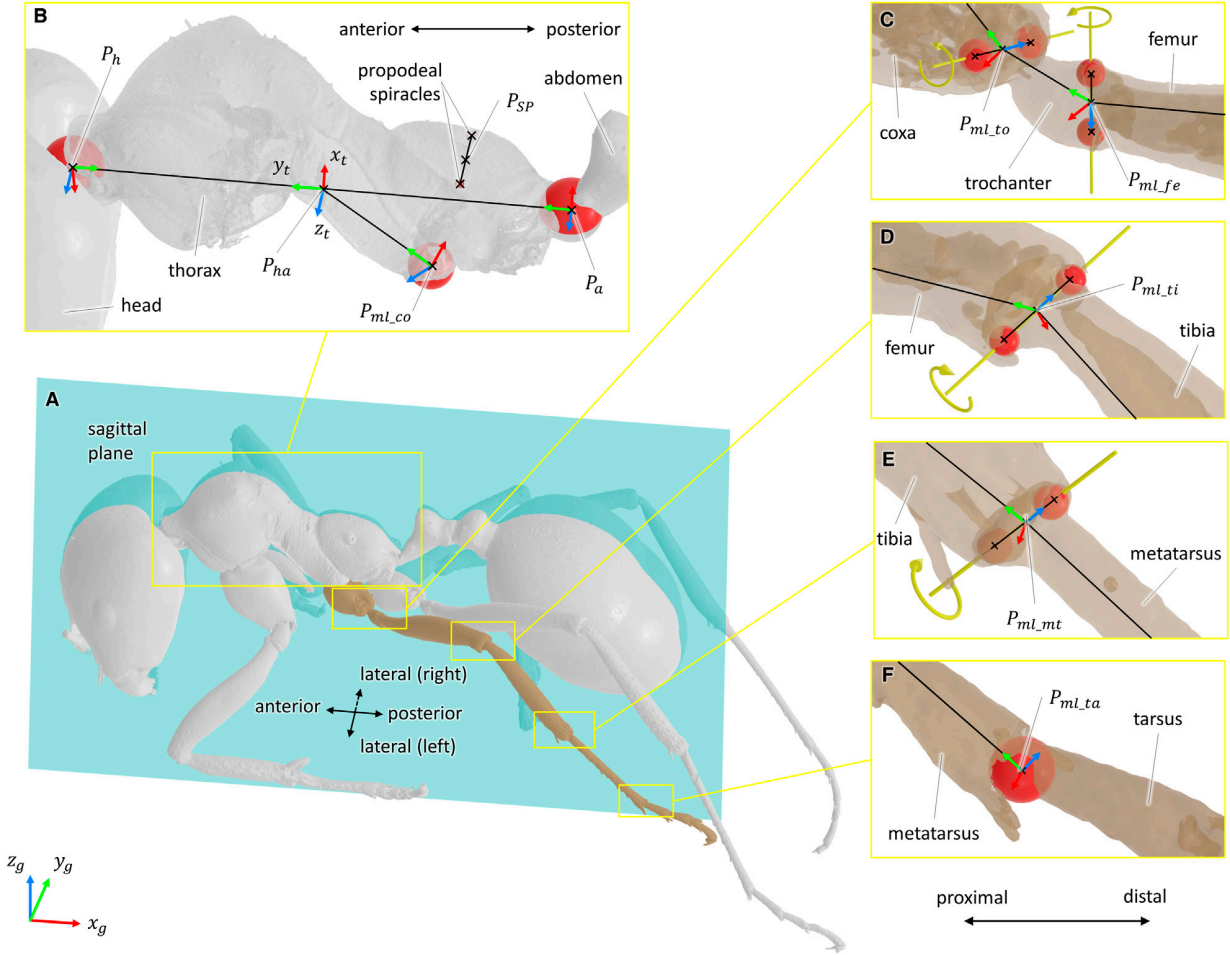
Definition of the joint geometrical parameters and coordinate systems. For all coordinate systems, the *x*-axis is represented in red, the *y*-axis in green, and the *z*-axis in blue. For ball-and-socket joints, a sphere fitted to the articular surface was considered as the center of the rotation of the joint. For hinge joints, the rotation axis was defined as the line passing through the center of two spheres fitted to the condyles of the joint. Fitted spheres are represented in red, and rotation axes are represented in yellow. **(A)** Representation of the sagittal plane. **(B)** Geometrical elements used to define the sagittal plane, the coordinate system of the thorax (*x*_*t*_, *y*_*t*_, *z*_*t*_), and the coordinate system of the middle left coxa. The point *P*_*SP*_ was defined as the mid-point of the line segment passing through the center of the two propodeal spiracles. The points *P*_*h*_ and *P*_*a*_ correspond to the center of the spheres fitted to the thorax/head and thorax/abdomen joints, respectively. The point *P*_*ml*_*co*_ corresponds to the center of the sphere fitted to the articular surface of the middle left thorax/coxa joint. **(C**–**E)** Geometrical elements used to define the rotation axes and the coordinate systems of the coxa/trochanter, trochanter/femur, femur/tibia, and tibia/metatarsus joints. In respective order, the points *P*_*ml*_*to*_, *P*_*ml*_*fe*_, *P*_*fe*_*ti*_, and *P*_*ti*_*mt*_ were defined as the mid-points of the line segments representing the rotation axis of the joints. **(F)** Geometrical elements used the rotation center and the coordinate systems of the metatarsus/tarsus joint. The point *P*_*ml*_*ta*_ corresponds to the center of the sphere fitted to the articular surface of the metatarsus/tarsus joint.

Defining the types of joints was done from both literature and morphometric data (Liu et al., 2019). From the 3D models of the body segments, joint geometrical parameters were estimated from the articular surfaces of the exoskeleton using a CAD software (3D EXPERIENCE, Dassault Systèmes, France). For ball-and-socket joints, the center of a sphere fitted to the articular surface was considered as the center of rotation of the joint (see Figures 2B,F). For hinge joints, the rotation axis was defined as the line passing through the center of two spheres fitted to the condyles of the joint (see Figures 2C–E). The procedure to determine joint geometrical parameter was also used in insect biomechanical modeling by Blanke et al. (2017). Because of low perceived motion and to facilitate the convergence of the inverse kinematics algorithm, the internal rotation of the metatarsus of each leg was not considered [it was assumed as a blocked degree of freedom (DOF)].

### 2.4 Creation of the Multibody Model

A multibody model was created, representing the whole-body locomotor system of the *Messor barbarus*.

According to the recommendations of the ISB (Wu et al., 2002, 2005), a coordinate system was defined for each body segment and for the ground. All coordinate systems were defined as right-handed and orthogonal, as follows (see Figure 2):

• Definition of the sagittal plane: plane perpendicular to the line passing through the center of two spheres fitted to the propodeal spiracles and containing the point *P*_*SP*_. Point *P*_*SP*_ was defined as the mid-point of the line segment defined by the two propodeal spiracles, see Figure 2B.
• Global coordinate system (*x*_*g*_, *y*_*g*_, *z*_*g*_): The *z*_*g*_-axis points upward, parallel to the field of gravity. The *x*_*g*_-axis points in the direction opposite the direction of travel. The *y*_*g*_-axis was defined as the common axis perpendicular to *x*_*g*_- and *z*_*g*_-axes.
• Thorax coordinate system (*x*_*t*_, *y*_*t*_, *z*_*t*_): the origin of this coordinate system was defined as the mid-point of the line segment passing through the center of the spheres fitted to the thorax/neck joint and thorax/abdomen joints, points *P*_*h*_ and *P*_*a*_ in Figure 2B, respectively. The *y*_*t*_-axis was defined parallel to the line segment *P*_*h*_ *P*_*a*_ and pointing anteriorly. The *x*_*t*_-axis was defined as the common axis perpendicular to the normal vector of the sagittal plane and to *y*_*t*_. The *z*_*t*_-axis was defined as the common axis perpendicular to *x*_*t*_- and *y*_*t*_-axes.
• For hinge joints, the origin of the coordinate system was chosen as the mid-point of the line segment representing the rotation axis (for example, points *P*_*ml*_*to*_ and *P*_*ml*_*fe*_ in Figure 2C, *P*_*ml*_*ti*_ in Figure 2D, and *P*_*ml*_*mt*_ in Figure 2E). The *z*-axis was defined parallel to the rotation axis and pointing medially. The *y*-axis was defined perpendicular to the *z*-axis and pointing to the origin of the coordinate system of the previous segment. The *x*-axis was defined as the common axis perpendicular to *y*- and *z*-axes.
• For ball-and-socket joints, the origin of the coordinate system was chosen as the center of the sphere fitted to the articular surface (for example, points *P*_*ml*_*co*_ and *P*_*ml*_*ta*_ in Figures 2B,F, respectively). The *y*-axis was defined parallel to the line passing through the origin of the coordinate system and the origin of the coordinate system of the previous segment and pointing proximally. The *x*-axis was defined as the common axis perpendicular to the normal vector of the sagittal plane and to *y*. The *z*-axis was defined as the common axis perpendicular to *x*- and *y*-axes.

According to the previous definitions of the coordinate systems, the following convention for rotations was adopted: abduction, positive rotation about the *x*-axis; adduction, negative rotation about the *x*-axis; internal rotation, positive rotation about the *y*-axis; external rotation, negative rotation about the *y*-axis; flexion, negative rotation about the *z*-axis; and extension, positive rotation about the *z*-axis.

**FIGURE 3:**
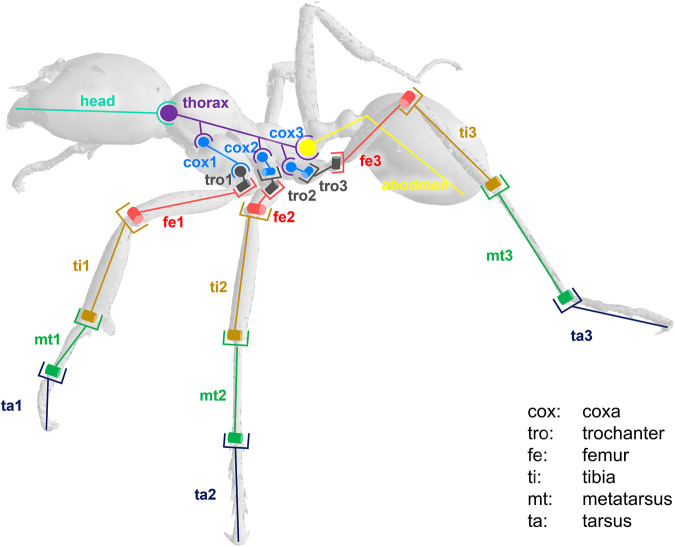
Kinematic chain representing half of the ant locomotor system. Anatomy: th (thorax), pet (petiole), abd (abdomen), cox (coxa), tro (trochanter), fe (femur), ti (tibia), mt (metatarsus), ta (tarsus). Type of joint: hinge (example: mt/ta) or ball and socket (example: head/thorax).

**FIGURE 4:**
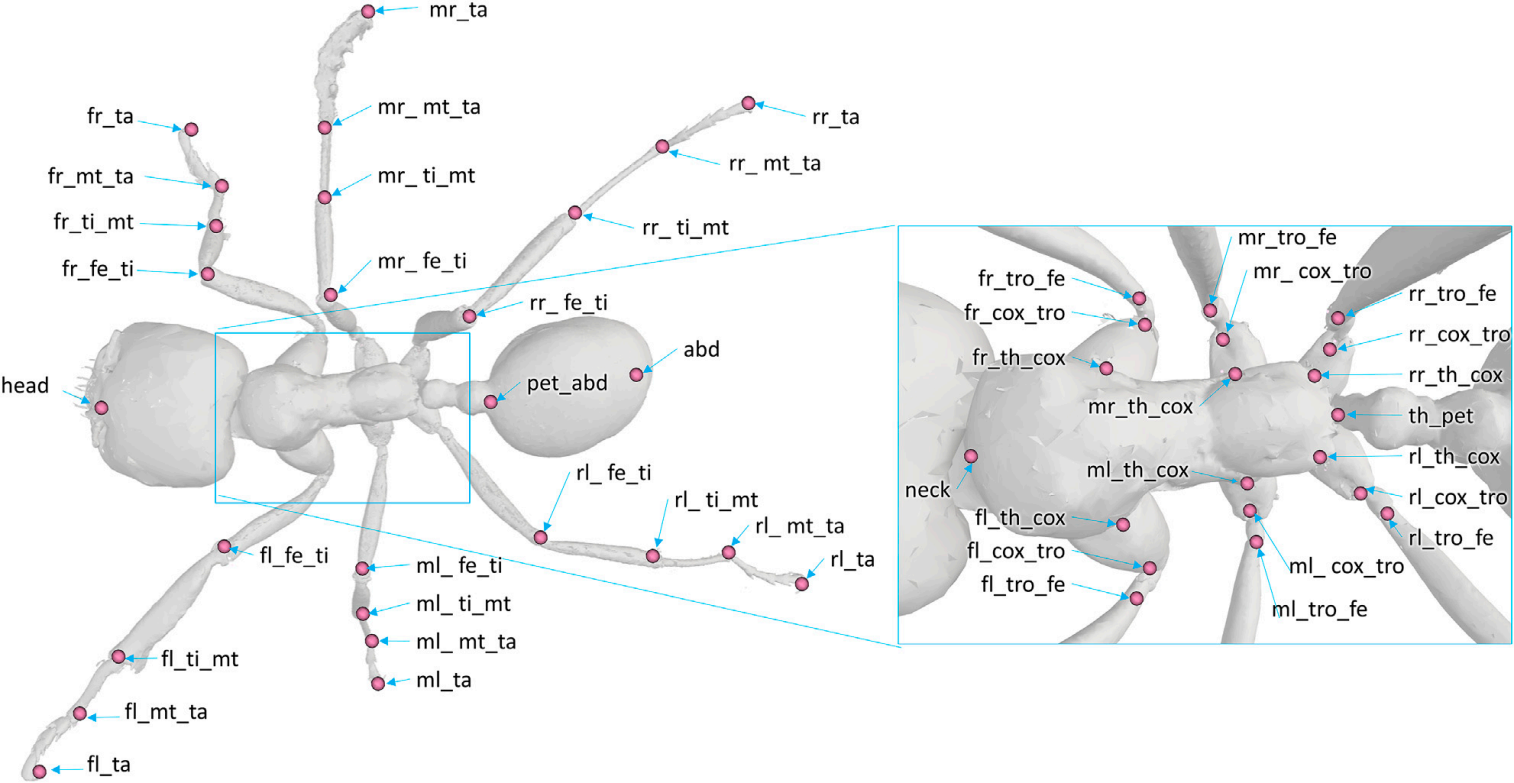
Position and denomination of the markers on the model. The following abbreviations are used for the denomination of the markers. Prefix: f (front), m (middle), or r (rear); position: l (left), r (right). Anatomy and articulation: th (thorax), pet (petiole), abd (abdomen), cox (coxa), tro (trochanter), fe (femur), ti (tibia), mt (metatarsus), ta (tarsus).

The model was composed of 39 segments and 65 DOFs. Segments were considered as rigid bodies, and joints were considered without clearance. Half of the kinematic chain of this model is presented in Figure 3. Forty-seven virtual markers were placed on the model according to the tracked anatomical landmarks (see Figure 4). The model was created using the software tool NSM Builder (version 2.1) (Valente et al., 2017) and finally exported in an OpenSim format. The range of motion of the joints was constrained to feasible values to aid the convergence of the inverse kinematics algorithm. These values were determined in OpenSim by articulating each DOF of the model until some structures of the joint segments touch each other. Obtained values are presented in Tables 1 and 2.

**TABLE 1:** Maximum range of motion allowable for each degree of freedom of trunk joints. Values are presented per leg: front, middle, and rear. The same values were used for left and right legs. These values were determined in OpenSim by articulating each degree of freedom of the model until some structures of the joint segments touch each other.

degree of freedom	Maximum allowable range of motion (deg)
thorax/head adduction	75
thorax/head internal rotation	40
thorax/head flexion	120
thorax/abdomen adduction	25
thorax/abdomen internal rotation	60
thorax/abdomen flexion	100

**TABLE 2:** Maximum range of motion allowable for each degree of freedom of the leg joints. Values are presented per leg: front, middle, and rear. The same values were used for left and right legs. These values were determined in OpenSim by articulating each degree of freedom of the model until some structures of the joint segment touch. Non-allocated values (NA) correspond to blocked degrees of freedom.

degree of freedom	Front legs (deg)	Middle legs (deg)	Rear legs (deg)
thorax/cox abduction	70	80	110
thorax/cox internal	40	55	105
thorax/cox flexion	80	100	105
cox/tro abduction	120	NA	NA
cox/tro internal	165	NA	NA
cox/tro flexion	180	120	130
tro/fe flexion	130	180	120
fe/ti flexion	160	165	190
ti/mt flexion	190	200	175
mt/ta flexion	200	200	240

### 2.5 Kinematic Data Acquisition and Treatment

**FIGURE 5:**
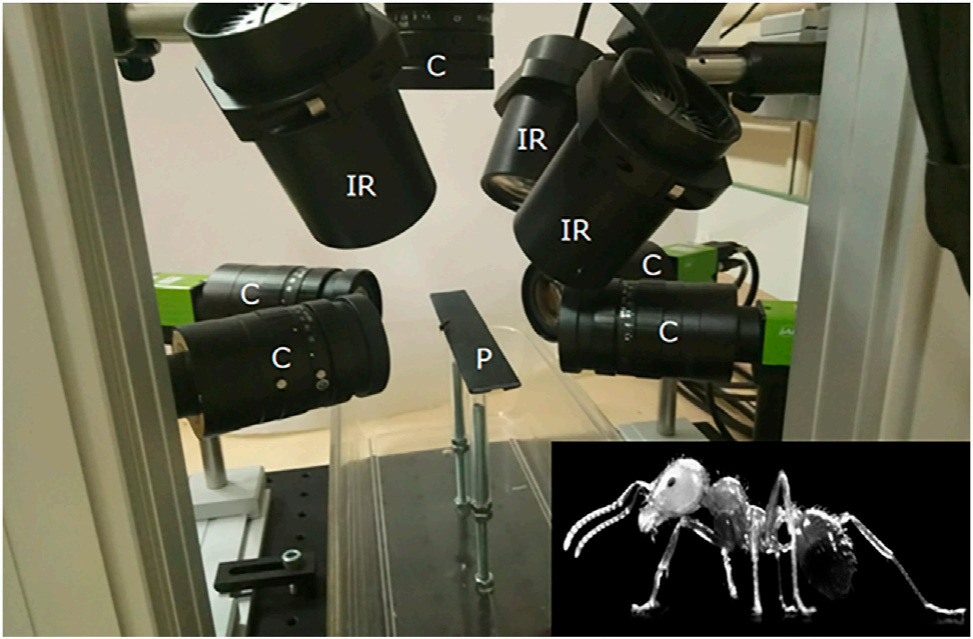
Video acquisition system. The experimental setup was composed of a wide walkway where the ant walked through and was captured by five cameras. C: cameras, IR: infrared spots, P: 250 × 20 mm wide walkway.

Kinematic data of a free walking ant (mean speed over the length of the calibrated walkway: 3.4 mm s^−1^) were acquired using high-speed synchronized video cameras (AI GO-5000M-PMCL). The experimental setup was composed of a wide walkway where the ant walked through, with five cameras (one on the top and two for each side or the walkway) and three infrared spots (see Figure 5). The shutter time was 1/3,333 s, and the acquisition time was set to 10 s with a sampling frequency of 300 Hz. The infrared spots were added to compensate this short shutter time. The resolution of the camera sensor was 2,560 × 2,048 pixels. Using the Hiris software of R&D Vision (version 5.2.0), the active sensor window was adjusted to the ant size in a 2,000 × 418 pixel rectangular area. The average field of vision of the cameras was 15.8 × 4.9 × 7.8 mm that gives a spatial resolution of 0.0096 mm/pixel. Obtained raw videos are available from the project repository.

Following a similar protocol as Merienne et al. (2020), the filming procedure was as follows. (1) The ant was randomly collected from the colony and left in a box for 15 min in order to reduce the stress of the capture. (2) The ant was located at the beginning of the walkway and the recording started when it entered in the calibrated volume. The temperature of the room was 26 ± 0.2° during the filming procedure. Only one gait cycle was studied to avoid the variability of the motor control during different gait cycles (change of the walking speed, balance management, and change of movement direction).

Video recordings were processed afterwards with the Vicon Peakmotus (version 10) software tool. Segment extremities were tracked semi-automatically during a gait cycle using a pattern-matching technique. The gait cycle was defined when the left middle leg leaves the ground and lifts, and it ends when that same leg leaves the ground again. Kinematic data were filtered with fourth-order Butterworth low-pass filters with a cutoff frequency of 5 Hz. It was then resampled from 300 to 100 Hz to decrease computation time. Spatial coordinates of the anatomical landmarks (those represented in Figure 4) were exported on a c3d format file. This file is available from the project repository.

### 2.6 Model Scaling and Inverse Kinematics Analysis

Spatial coordinates of the anatomical landmarks were used to scale the multibody model and to run inverse kinematics simulations. A scaling procedure was carried out to fit the model (originally created from the morphology of specimen 1) to the morphology of specimen 2. This was performed using the open-source software tool OpenSim (version 4.0) (Seth et al., 2018). Using the scaled model, inverse kinematics simulations were also performed in OpenSim. Joint angles as well as root mean square errors (RMSEs) were obtained from these simulations.

### 2.7 Propagation of Model Parameter Uncertainties

In order to evaluate the sensitivity of the calculated kinematic data to model parameter uncertainties, two MC simulations were conducted. A similar procedure was used by Martelli et al. (2015) and Myers et al. (2015).

In the first MC simulation, the position of model markers was randomly perturbed according to their uncertainty. Random values were assumed to have a uniform distribution (i.e., all outcomes were considered as equally likely). Variations were assumed to be the same in all directions of the measurement volume. Therefore, the uncertainty zone for the model markers was assumed to be spherical. The radius of these spherical uncertainty zones was chosen as a common residual value for the camera calibration process for the used experimental setup: 0.4 mm.

In the second MC simulation, joint geometrical parameters (location and orientation) were randomly disturbed. The uncertainty in location and orientation of joints is mainly related with operator-dependent variability of the treatment and identification of the articular surfaces. In order to define perturbation magnitude (translation and rotation) introduced to the joint geometrical parameters, several procedures of identification of articular surfaces were carried out. Cylindrical uncertainty zones were assumed for hinge joints, while spherical uncertainty zones were assumed for ball-and-socket joints. The radius of the cylindrical and spherical uncertainty zones was considered to be the same for all the joints and equal to 0.2 mm.

**FIGURE 6:**
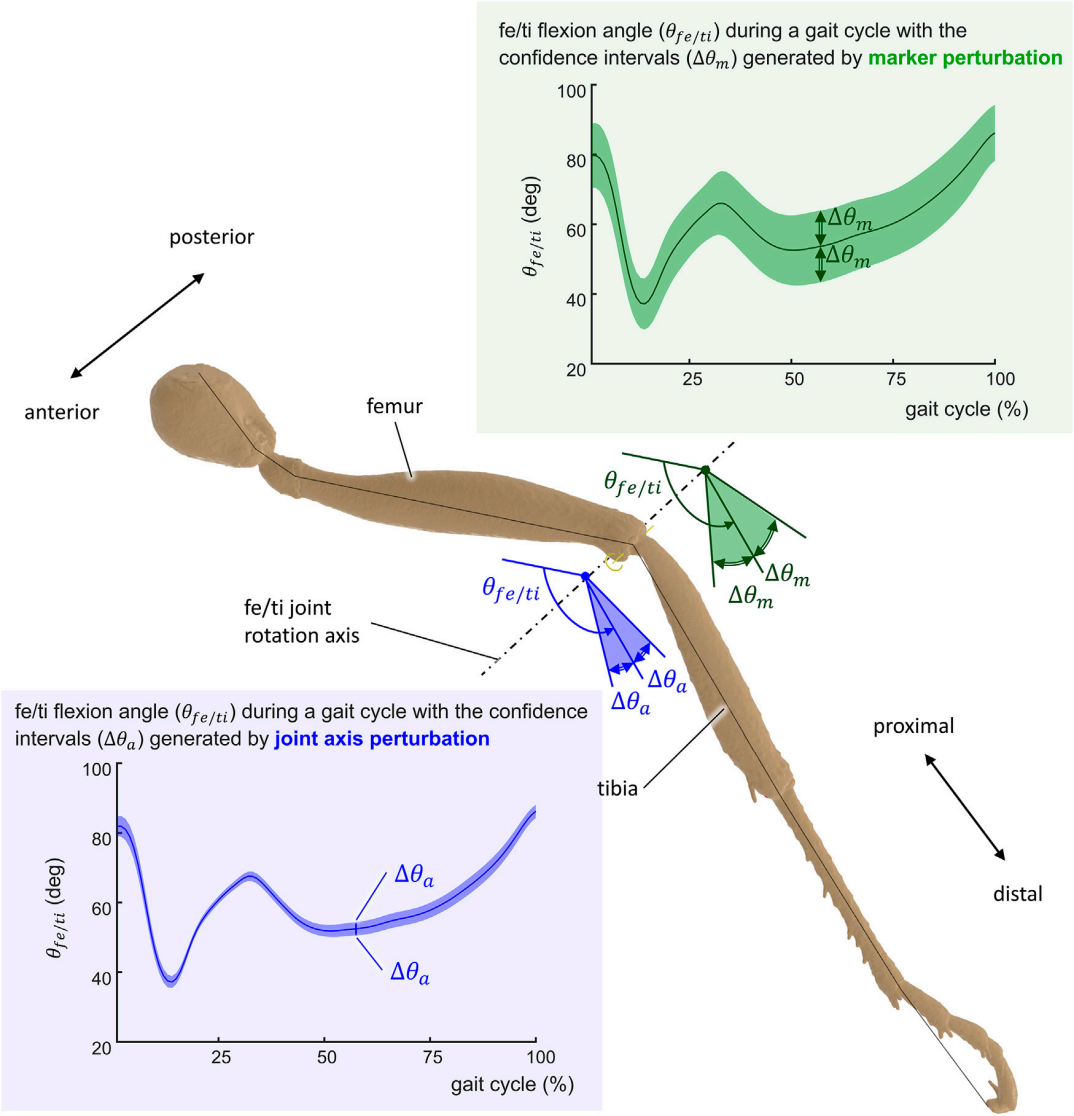
Representation of the uncertainty of the computed kinematics of the ant gait. The uncertainty Δ*θ* is represented for the femur/tibia angle (*θ*_*fe*/*ti*_) of the middle left leg for joint axis perturbation Δ*θ*_*a*_ (blue-shaded confidence intervals) and marker position perturbation Δ*θ*_*m*_ (green-shaded confidence intervals).

These MC simulations were implemented and run by means of the OpenSim API. One thousand iterations were carried out for each MC simulation, which were enough to guarantee a stabilization of average values. Average values of joint angles at each time step were calculated from the obtained results. Coverage intervals were defined as twice the standard deviation. A graphical representation of these results is presented in Figure 6. The sensitivity of the kinematic results regarding model parameter uncertainties was defined as the signal-to-noise ratio (SNR) of the joint angles during the gait. The SNR was calculated as the maximum amplitude of the signal (also called power of the signal, *P*_*s*_) divided by the maximum coverage interval (also called power of the noise, *P*_*n*_) of the joint angle during the gait. Therefore, an SNR value was obtained per degree of freedom for the analyzed gait cycle.

## 3 Results

In order to determine how modeling assumptions affect inverse kinematic results at the ant scale, a multibody model of the *Messor barbarus* was developed together with a simulation framework to evaluate its sensitivity. Both the model and the simulation framework are freely available on the SimTK repository: https://simtk.org/projects/barbarus. From the experimental kinematic data, an inverse kinematic simulation was conducted. The results of this simulation, representing a gait cycle of free locomotion of the *Messor barbarus*, are summarized in Tables 3 and 4. A video of the simulated kinematics is available from the project repository.

**TABLE 3:** Results of the inverse kinematic simulation for the trunk joints. Reported values represent the range of motion in degrees of the joint angles.

degree of freedom	Range of motion (deg)
thorax/head abduction	19.5
thorax/head internal rotation	9.0
thorax/head flexion	13.3
thorax/abdomen abduction	11.9
thorax/abdomen internal rotation	14.2
thorax/abdomen flexion	13.8

**TABLE 4:** Results of the inverse kinematic simulation for the leg joints. Reported values represent the range of motion in degrees of the joint angles. Non-allocated values (NA) correspond to blocked degrees of freedom.

degree of freedom	Front right leg (deg)	Middle right leg (deg)	Rear right leg (deg)	Front left leg (deg)	Middle left leg (deg)	Rear left leg (deg)
thorax/cox abduction	40.7	32.0	16.5	19.1	23.4	27.2
thorax/cox internal rotation	29.3	54.0	35.7	28.1	26.0	16.3
thorax/cox flexion	26.5	25.9	90.6	22.1	39.4	14.9
cox/tro abduction	78.2	NA	NA	74.6	NA	NA
cox/tro internal rotation	76.3	NA	NA	47.3	NA	NA
cox/tro flexion	90.0	30.6	30.2	43.9	48.3	42.4
tro/fe flexion	120.7	53.1	103.9	78.6	27.2	15.6
fe/ti flexion	61.6	73.6	66.7	39.3	38.2	60.2
ti/mt flexion	53.8	41.0	29.0	63.4	17.7	17.2
mt/ta flexion	56.9	36.2	43.5	57.7	NA	23.4

These results correspond to the range of motion of the joint angles. The whole set of results is available from the project repository and can also be reproduced from the model and the experimental kinematic data. It can be noticed that the trochanter/femur (tr/fe) joint is the one with the wider range of motion, while the thorax/coxa joints exhibit the smallest one. The average RMSE of the inverse kinematic simulation was 0.21 mm, which corresponds to 3.2% of the specimen size.

The sensitivity of the kinematic results regarding model parameter uncertainties was evaluated by means of the SNR. These results are summarized per set of joints, from marker perturbation as well as from axis perturbation, in Table 5. High SNR values indicate that the power of the signal (computed joint angle) is representative with respect to the power of the noise (confidence intervals). SNR values near or lower than 1 indicate that the dynamics of the signal of interest might be hidden by noise. It can be noticed that the computed kinematics is more sensitive to marker perturbation compared to joint axis perturbation (Table 5). The perturbation applied to the markers generated an SNR of 2.2 in average for all the joints. This means that the dynamics of the studied signal (computed joint angles) can be observed despite possible variations during the motion analysis process. The SNR from axis perturbation was almost four times higher than that from marker perturbation. No significant differences in sensitivity were found between the joints of the legs on the right side of the body with respect to those on the left side. No tendency can be inferred from the sensitivity of the joints with respect to their anterior–posterior position: front, middle, and rear. The joint that showed the highest SNR values (consequently a lower sensitivity) was the fe/ti joint, and this was the case for both marker and axis perturbations.

**TABLE 5:** Results of the sensitivity analysis. These results represent the average signal-to-noise ratio per joint obtained from marker perturbation (first column) and axis perturbation (second column). In the case of cox/tro, tro/fe, fe/ti, ti/mt, and mt/ta joints, averages were calculated from the signal-to-noise ratio of the six legs.

Joint	Signal-to-noise ratio from marker perturbation	Signal-to-noise ratio from axis perturbation
all joints	2.20	8.10
right-hand side joints	2.29	7.96
left-hand side joints	2.27	8.92
front legs joints	1.54	9.13
middle legs joints	2.82	8.05
rear legs joints	2.69	7.97
thorax/head	1.71	5.08
thorax/abdomen	1.38	4.97
thorax/cox	1.58	4.65
cox/tro	2.73	7.25
tro/fe	1.57	5.52
fe/ti	6.87	27.63
ti/mt	1.70	8.62
mt/ta	1.19	7.14

**FIGURE 7:**
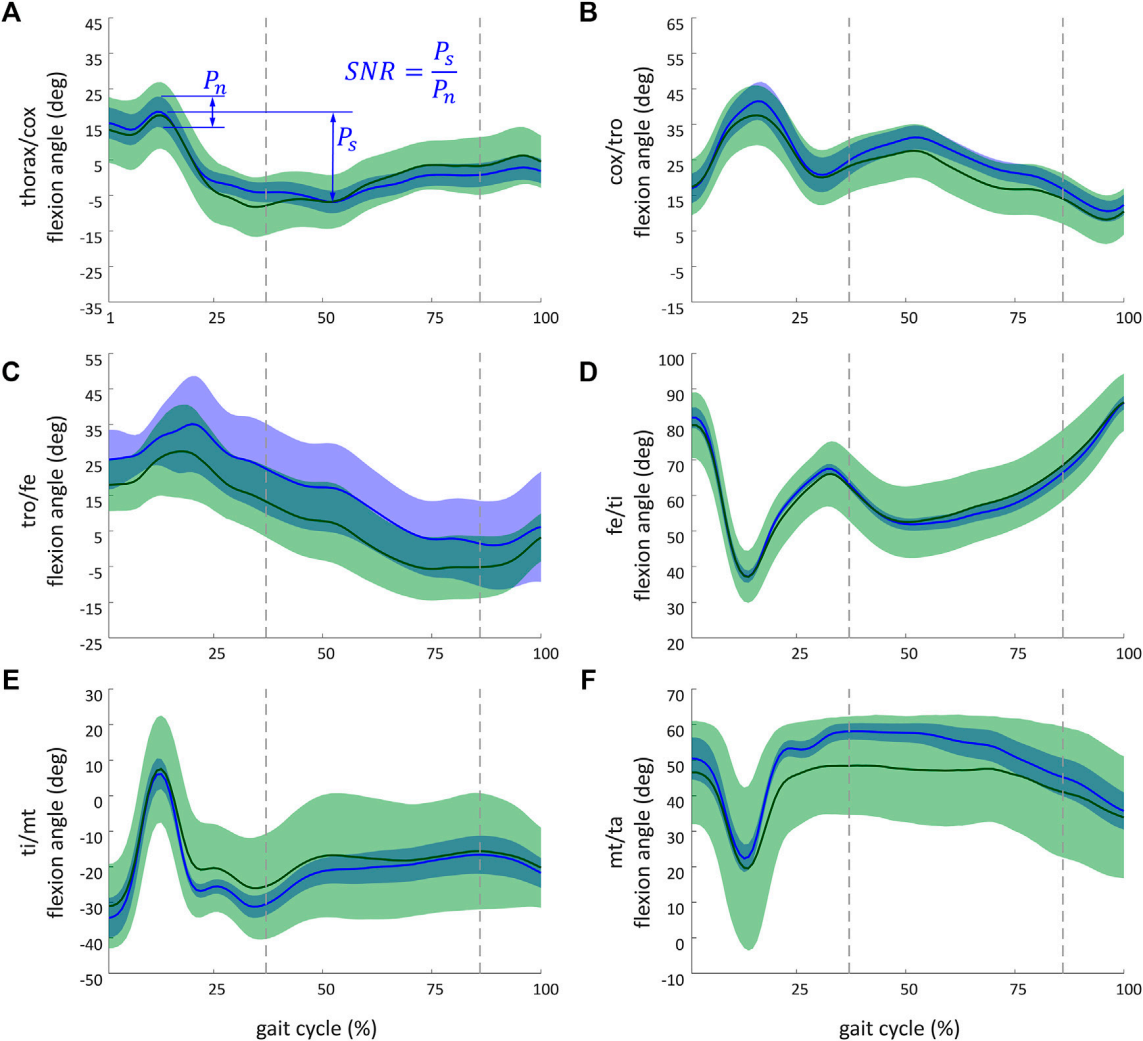
Kinematic results obtained from the simulation of the ant model and the experimental kinematic data for flexion angle of the middle right leg of **(A)** thorax/cox; **(B)** cox/tro; **(C)** tro/fe; **(D)** fe/ti; **(E)** ti/mt, and **(F)** mt/ta. The recorded and simulated gait cycles lasted 1.39 s. These results are a sample of the whole set of results available from the project repository. Solid lines indicate the mean values from the Monte Carlo simulations from the marker perturbation (green) and from the axis perturbation (blue). For marker and axis perturbations, respectively, the green- and blue-shaded regions represent the confidence interval (calculated as twice the standard deviation). The dashed vertical lines (37% and 87%) indicate when legs of both tripods were on the ground. The SNR of the thorax/cox flexion angle obtained from the axis perturbation simulation is illustrated in **(A)**. *P*_*s*_ (standing for power of the signal) corresponds to the peak-to-peak amplitude of the signal. *P*_*n*_ (standing for power of the noise) corresponds to the maximal coverage interval of the joint angle during the gait.

Figure 7 illustrates kinematic results obtained from the simulation of the ant model and the experimental kinematic data for joints of the middle right leg [(Figure 7A) thorax/cox, (Figure 7B) cox/tro, (Figure 7C) tro/fe, (Figure 7D) fe/ti, (Figure 7E) ti/mt, and (Figure 7F) mt/ta flexion angles]. Mean values (in solid lines) from both MC simulations (marker and axis perturbations) are shown with their corresponding confidence intervals (shaded regions). The green line and shaded region represent the results from the marker perturbation, and the blue line and shaded region represent the results from the axis perturbation. The SNR of the thorax/cox flexion angle obtained from the axis perturbation simulation is illustrated in Figure 7A. From these results, it can be noticed that the confidence intervals of the joint angles when disturbing the axis location and orientation were smaller than the confidence intervals obtained from the marker position perturbation.

## 4 Discussion and Conclusions

In this paper, the propagation of parameter uncertainties in kinematic modelling has been evaluated at the small scale. This work demonstrates the feasibility of using biomechanical models to study locomotion in relatively small animals. Because of their scale, motion analysis techniques for hexapods are less developed compared to those for quadrupeds and bipeds. In relatively big animals, the use of several reflective markers per segment allows a good precision of the kinematic data. However, the use of physical markers is not easy in motion analysis in small insects. This implies that the capabilities of the small-scale biomechanical modeling techniques must be well evaluated.

To do so, a multibody model of a *Messor barbarus* ant was developed. It is available in open source from the project repository and can be used and enhanced by the scientific community. Besides, the model could allow biologists to study function/structure relationships of *Messor barbarus*. The whole set of experimental and simulated kinematic data is also available from the project repository.

In spite of the differences in morphology of the studied species, the obtained joint angles were in the same order of magnitude as those reported in the literature about ant kinematics (see Table 6). The difference between angle range of left and right legs comes from the fact that the ant did not walk perfectly straight. Obtained kinematic data are valuable for roboticians to implement bio-inspired gaits in robots (see Ouyang et al. (2021) for example).

**TABLE 6:** Summary of studies investigating ant kinematics.

Study	Analyzed angle	Specie	Methods	Range of motion (deg)	Corresponding range of motion from this study (deg)
Weihmann and Blickhan (2009)	thorax/head flexion	*Cataglyphis fortis*	video-based analysis (250 Hz, 480 × 480 pixels of camera resolution)	5	13
Weihmann and Blickhan (2009)	thorax/head flexion	*Formica pratensis*	video-based analysis (250 Hz, 480 × 480 pixels of camera resolution)	5	13
Reinhardt and Blickhan (2014)	thorax/head flexion	*Formica polyctena*	video-based analysis (500 Hz, 768 × 512 pixels of camera resolution)	10	13
Weihmann and Blickhan (2009)	thorax/abdomen flexion	*Cataglyphis fortis*	video-based analysis (250 Hz, 480 × 480 pixels of camera resolution)	10	14
Weihmann and Blickhan (2009)	thorax/abdomen flexion	*Formica pratensis*	video-based analysis (250 Hz, 480 × 480 pixels of camera resolution)	14	14
Reinhardt and Blickhan (2014)	thorax/abdomen flexion	*Formica polyctena*	video-based analysis (500 Hz, 768 × 512 pixels of camera resolution)	10	14
Guo et al. (2018)	thorax/cx flexion angle on middle left leg	*Cataglyphis fortis*	video-based analysis (500 Hz, 480 × 480 pixels of camera resolution)	63	39
Guo et al. (2018)	cx/fe flexion angle on middle left leg	*Cataglyphis fortis*	video-based analysis (500 Hz, 480 × 480 pixels of camera resolution)	37	27
Guo et al. (2018)	fe/tb flexion angle on middle left leg	*Cataglyphis fortis*	video-based analysis (500 Hz, 480 × 480 pixels of camera resolution)	83	38

A possible error source in the conducted kinematic simulation could be linked to the use of two different specimens for acquiring experimental data (one for the geometrical 3D model and one for the experimental kinematic data). When using two subjects to perform a constrained kinematics simulation, a scaling procedure is required, which is naturally an additional source of errors. This might be one of the main reasons for the obtained RMSE values. In comparison to human locomotion simulations, the obtained normalized RMSE values for ant locomotion simulation were greater. In human simulations, it is recommended not to exceed 0.6% relative RMSE regarding body size (in contrast to a normalized RMSE of 3.2% obtained in this work). This difference can also be related to the fact that the ant body is composed of more segments than the human one.

Thanks to the developed model, the impact of the propagation of model parameter uncertainties in inverse kinematic simulations at the insect scale was evaluated. Obtained SNR values indicate that the geometric and kinematic measurement techniques used are feasible for the development of multibody models at the ant scale. The fact that the model is more sensitive to marker perturbations indicates that efforts in kinematic modeling at the ant scale must be centered around the kinematic acquisition (marker definition, placement, tracking, etc.) rather than geometric acquisition (µ-CT, segmentation, joint parameter definition, etc.). The fact of experiencing lower sensitivity at the fe/ti joint can be explained by the large range of motion of this joint and, also, because it is composed of the two longest segments of the limb. Long segments are easier to track, plus the perturbation of the measurement process has a lower impact than in the case of short segments. The fact of having no significant differences in sensitivity between the joints of the legs on the right side of the body compared to those on the left side can be associated to the symmetry of the video acquisition system regarding the walkway.

This study presents several limitations, however. From an experimental point of view, the following aspects can be improved. Each body segment was tracked by only two markers. The number of tracked markers per segments could be increased to improve the quality of the simulation. Additionally, emerging automatic tracking techniques (i.e., deep-learning-powered motion tracking) must be explored as an alternative to reduce tracking time and to increase the number of tracked points per segment. Finally, the four tarsal segments were all lumped into a unique rigid segment. This was due to the configuration and the capacity of the experimental setup (camera resolution, number of cameras, camera position, etc.), which did not provide enough resolution to track the tarsal segments individually. On the other hand, from a modeling point of view, the segments of the ant were considered as rigid bodies because of the complexity of taking body deformation into consideration. This assumption merits a profound analysis in order to determine the effects of segment compliance in insect locomotion, which seems to play an important role (Blickhan et al., 2021).

Finally, future work is required to develop a dynamic model of the ant gait. This requires determining muscle parameters (geometrical and force-generating parameters), segment mass and inertia properties, and ground reaction forces. This study contributes to the construction of a musculoskeletal model of ants which can be useful in the study of evolution, neural control, and biomimetic applications.

## Data Availability

The datasets presented in this study can be found in online repositories. The names of the repository/repositories and accession number(s) can be found in the article/Supplementary Material.
